# *N*′-(2-Hydroxy­benzyl­idene)-4-methoxy­benzohydrazide

**DOI:** 10.1107/S1600536808008088

**Published:** 2008-03-29

**Authors:** Chun-Bao Tang

**Affiliations:** a Department of Chemistry, Jiaying University, Meizhou 514015, People’s Republic of China

## Abstract

The title Schiff base compound, C_15_H_14_N_2_O_3_, was derived from the condensation reaction of salicylaldehyde with 4-methoxy­benzohydrazide. The dihedral angle between the two benzene rings is 2.5 (2)°. In the crystal structure, mol­ecules are linked through inter­molecular N—H⋯O hydrogen bonds, forming chains running along the *b* axis.

## Related literature

For related structures, see: Tang (2006 ▶, 2007*a* ▶,*b* ▶,*c* ▶,*d* ▶). For reference structural data, see: Allen *et al.* (1987 ▶).
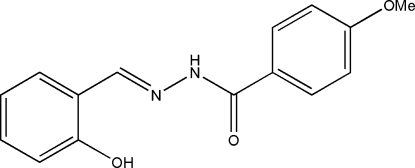


## Experimental

### 

#### Crystal data

C_15_H_14_N_2_O_3_
*M* *_r_* = 270.28
Monoclinic, 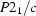

*a* = 16.283 (4) Å
*b* = 5.1876 (12) Å
*c* = 16.303 (4) Å
β = 108.093 (2)°
*V* = 1309.0 (5) Å^3^
*Z* = 4
Mo *K*α radiation
μ = 0.10 mm^−1^
*T* = 298 (2) K
0.23 × 0.20 × 0.17 mm

#### Data collection

Bruker SMART CCD area-detector diffractometer
Absorption correction: multi-scan (*SADABS*; Sheldrick, 1996 ▶) *T*_min_ = 0.978, *T*_max_ = 0.984
7166 measured reflections
2862 independent reflections
2288 reflections with *I* > 2σ(*I*)
*R*_int_ = 0.024

#### Refinement

*R*[*F* ^2^ > 2σ(*F* ^2^)] = 0.038
*wR*(*F* ^2^) = 0.111
*S* = 1.03
2862 reflections
183 parameters
H-atom parameters constrained
Δρ_max_ = 0.18 e Å^−3^
Δρ_min_ = −0.14 e Å^−3^

### 

Data collection: *SMART* (Bruker, 2002 ▶); cell refinement: *SAINT* (Bruker, 2002 ▶); data reduction: *SAINT*; program(s) used to solve structure: *SHELXS97* (Sheldrick, 2008 ▶); program(s) used to refine structure: *SHELXL97* (Sheldrick, 2008 ▶); molecular graphics: *SHELXTL* (Sheldrick, 2008 ▶); software used to prepare material for publication: *SHELXL97*.

## Supplementary Material

Crystal structure: contains datablocks global, I. DOI: 10.1107/S1600536808008088/sj2477sup1.cif

Structure factors: contains datablocks I. DOI: 10.1107/S1600536808008088/sj2477Isup2.hkl

Additional supplementary materials:  crystallographic information; 3D view; checkCIF report

## Figures and Tables

**Table 1:** Hydrogen-bond geometry (Å, °)

*D*—H⋯*A*	*D*—H	H⋯*A*	*D*⋯*A*	*D*—H⋯*A*
N2—H2⋯O2^i^	0.90	2.18	3.0112 (15)	153
O1—H1⋯N1	0.82	1.90	2.6171 (14)	146

Symmetry code: (i) 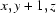.

**Fig. 1.**
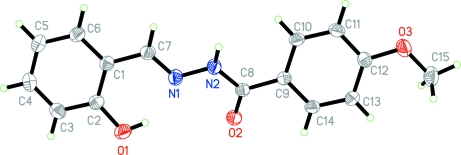
The molecular structure of the compound, showing the atom-numbering scheme. Displacement ellipsoids are drawn at the 30% probability level.

**Fig. 2.**
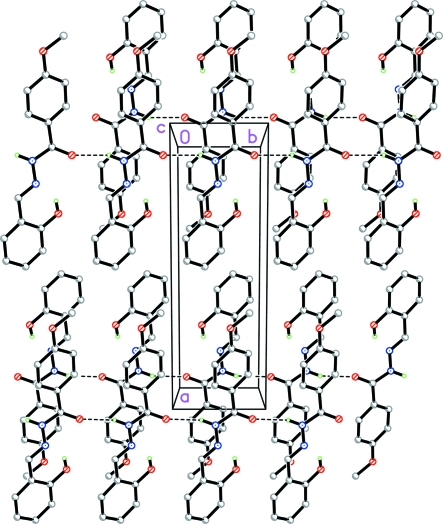
Molecular packing of (I) with hydrogen bonds drawn as dashed lines.

## supplementary crystallographic information

### Comment

Recently, the author has reported the structures of several Schiff base compounds (Tang, 2006, 2007*a*,*b*,*c*,*d*) and, in continuation of work in this area, reports herein the structure of the title compound, (I), Fig. 1, a new Schiff base compound.

In the title compound (Fig. 1), the dihedral angle between the two benzene rings is 2.5 (2)°. The torsion angles C1—C7—N1—N2, C7—N1—N2—C8, and N1—N2—C8—C9 are 1.3 (2), 11.4 (2), and 0.6 (2)°, respectively. All the bond lengths are within normal values (Allen *et al.*, 1987).

In the crystal structure of the compound, molecules are linked through N—H···O intermolecular hydrogen bonds (Table 1), forming chains running along the *b* axis (Fig. 2).

### Experimental

Salicylaldehyde (0.1 mmol, 12.2 mg) and 4-methoxybenzohydrazide (0.1 mmol, 16.6 mg) were dissolved in an ethanol solution (20 ml). The mixture was stirred at reflux for 10 min to give a clear colorless solution. Colorless needle-like crystals of the compound were formed by slow evaporation of the solvent over several days.

### Refinement

H atoms were constrained to ideal geometries, with C—H = 0.93–0.96 Å, O—H = 0.82 Å, N—H = 0.90 Å, and with *U*_iso_(H) = 1.2*U*_eq_(C,N), 1.5*U*_eq_(C15 and O1).

### Crystal data

**Table:**

C_15_H_14_N_2_O_3_	*F*_000_ = 568
*M**_r_* = 270.28	*D*_x_ = 1.371 Mg m^−^^3^
Monoclinic, *P*2_1_/*c*	Mo *K*α radiation λ = 0.71073 Å
Hall symbol: -P 2ybc	Cell parameters from 2841 reflections
*a* = 16.283 (4) Å	θ = 2.5–28.4º
*b* = 5.1876 (12) Å	µ = 0.10 mm^−^^1^
*c* = 16.303 (4) Å	*T* = 298 (2) K
β = 108.093 (2)º	Cut from a needle, colorless
*V* = 1309.0 (5) Å^3^	0.23 × 0.20 × 0.17 mm
*Z* = 4	

### Data collection

**Table:**

Bruker SMART CCD area-detector diffractometer	2862 independent reflections
Radiation source: fine-focus sealed tube	2288 reflections with *I* > 2σ(*I*)
Monochromator: graphite	*R*_int_ = 0.024
*T* = 298(2) K	θ_max_ = 27.0º
ω scans	θ_min_ = 2.6º
Absorption correction: multi-scan(SADABS; Sheldrick, 1996)	*h* = −20→17
*T*_min_ = 0.978, *T*_max_ = 0.984	*k* = −6→5
7166 measured reflections	*l* = −20→20

### Refinement

**Table:**

Refinement on *F*^2^	Hydrogen site location: inferred from neighbouring sites
Least-squares matrix: full	H-atom parameters constrained
*R*[*F*^2^ > 2σ(*F*^2^)] = 0.038	*w* = 1/[σ^2^(*F*_o_^2^) + (0.0543*P*)^2^ + 0.1695*P*] where *P* = (*F*_o_^2^ + 2*F*_c_^2^)/3
*wR*(*F*^2^) = 0.111	(Δ/σ)_max_ = 0.001
*S* = 1.03	Δρ_max_ = 0.19 e Å^−^^3^
2862 reflections	Δρ_min_ = −0.14 e Å^−^^3^
183 parameters	Extinction correction: SHELXL97 (Sheldrick, 2008), Fc^*^=kFc[1+0.001xFc^2^λ^3^/sin(2θ)]^-1/4^
Primary atom site location: structure-invariant direct methods	Extinction coefficient: 0.0102 (19)
Secondary atom site location: difference Fourier map	

### Special details

**Table:**

Geometry. All e.s.d.'s (except the e.s.d. in the dihedral angle between two l.s. planes) are estimated using the full covariance matrix. The cell e.s.d.'s are taken into account individually in the estimation of e.s.d.'s in distances, angles and torsion angles; correlations between e.s.d.'s in cell parameters are only used when they are defined by crystal symmetry. An approximate (isotropic) treatment of cell e.s.d.'s is used for estimating e.s.d.'s involving l.s. planes.
Refinement. Refinement of *F*^2^ against ALL reflections. The weighted *R*-factor *wR* and goodness of fit *S* are based on *F*^2^, conventional *R*-factors *R* are based on *F*, with *F* set to zero for negative *F*^2^. The threshold expression of *F*^2^ > σ(*F*^2^) is used only for calculating *R*-factors(gt) *etc*. and is not relevant to the choice of reflections for refinement. *R*-factors based on *F*^2^ are statistically about twice as large as those based on *F*, and *R*- factors based on ALL data will be even larger.

### Fractional atomic coordinates and isotropic or equivalent isotropic displacement parameters (Å^2^)

**Table:**

	*x*	*y*	*z*	*U*_iso_*/*U*_eq_	
O1	0.28687 (6)	0.79939 (18)	0.04446 (6)	0.0589 (3)	
H1	0.2415	0.8168	0.0558	0.071*	
O2	0.07668 (6)	0.61484 (17)	0.09720 (6)	0.0513 (3)	
O3	−0.28125 (6)	0.8712 (2)	0.15298 (7)	0.0603 (3)	
N1	0.18099 (6)	1.0151 (2)	0.11628 (7)	0.0458 (3)	
N2	0.09898 (6)	1.0412 (2)	0.12266 (7)	0.0458 (3)	
H2	0.0786	1.2025	0.1239	0.055*	
C1	0.32018 (7)	1.1824 (2)	0.13304 (8)	0.0418 (3)	
C2	0.34309 (8)	0.9849 (2)	0.08499 (8)	0.0447 (3)	
C3	0.42580 (9)	0.9783 (3)	0.07764 (9)	0.0539 (3)	
H3	0.4407	0.8497	0.0451	0.065*	
C4	0.48579 (9)	1.1612 (3)	0.11825 (9)	0.0564 (4)	
H4A	0.5410	1.1548	0.1129	0.068*	
C5	0.46509 (9)	1.3546 (3)	0.16693 (9)	0.0559 (4)	
H5	0.5063	1.4758	0.1950	0.067*	
C6	0.38275 (8)	1.3652 (3)	0.17323 (8)	0.0497 (3)	
H6	0.3685	1.4972	0.2050	0.060*	
C7	0.23383 (8)	1.2025 (2)	0.14075 (8)	0.0448 (3)	
H7	0.2172	1.3510	0.1634	0.054*	
C8	0.04904 (7)	0.8257 (2)	0.11109 (7)	0.0393 (3)	
C9	−0.03888 (7)	0.8543 (2)	0.11895 (7)	0.0376 (3)	
C10	−0.06242 (8)	1.0470 (2)	0.16743 (8)	0.0439 (3)	
H10	−0.0232	1.1763	0.1929	0.053*	
C11	−0.14321 (8)	1.0465 (2)	0.17758 (8)	0.0475 (3)	
H11	−0.1580	1.1738	0.2106	0.057*	
C12	−0.20273 (8)	0.8575 (2)	0.13885 (8)	0.0438 (3)	
C13	−0.18097 (8)	0.6676 (2)	0.08943 (8)	0.0465 (3)	
H13	−0.2209	0.5417	0.0626	0.056*	
C14	−0.09942 (8)	0.6672 (2)	0.08042 (8)	0.0436 (3)	
H14	−0.0847	0.5386	0.0478	0.052*	
C15	−0.33848 (9)	0.6588 (3)	0.12432 (11)	0.0682 (4)	
H15A	−0.3531	0.6430	0.0628	0.102*	
H15B	−0.3901	0.6869	0.1397	0.102*	
H15C	−0.3108	0.5034	0.1511	0.102*	

### Atomic displacement parameters (Å^2^)

**Table:**

	*U*^11^	*U*^22^	*U*^33^	*U*^12^	*U*^13^	*U*^23^
O1	0.0530 (6)	0.0498 (6)	0.0720 (6)	−0.0030 (4)	0.0166 (5)	−0.0180 (5)
O2	0.0492 (5)	0.0387 (5)	0.0677 (6)	0.0032 (4)	0.0206 (4)	−0.0064 (4)
O3	0.0521 (6)	0.0612 (6)	0.0769 (7)	−0.0039 (5)	0.0339 (5)	−0.0048 (5)
N1	0.0397 (5)	0.0411 (6)	0.0572 (6)	0.0003 (4)	0.0159 (5)	0.0006 (5)
N2	0.0392 (5)	0.0368 (6)	0.0628 (7)	0.0017 (4)	0.0180 (5)	−0.0017 (5)
C1	0.0423 (6)	0.0367 (6)	0.0455 (6)	−0.0002 (5)	0.0123 (5)	0.0029 (5)
C2	0.0471 (7)	0.0398 (7)	0.0455 (7)	0.0003 (5)	0.0118 (5)	0.0016 (5)
C3	0.0568 (8)	0.0536 (8)	0.0565 (8)	0.0054 (6)	0.0250 (6)	−0.0013 (6)
C4	0.0476 (7)	0.0623 (9)	0.0647 (8)	−0.0017 (6)	0.0253 (6)	0.0080 (7)
C5	0.0494 (7)	0.0522 (8)	0.0650 (8)	−0.0130 (6)	0.0163 (6)	0.0007 (7)
C6	0.0505 (7)	0.0422 (7)	0.0561 (8)	−0.0051 (6)	0.0163 (6)	−0.0040 (6)
C7	0.0451 (7)	0.0390 (7)	0.0499 (7)	0.0019 (5)	0.0141 (5)	−0.0020 (5)
C8	0.0421 (6)	0.0361 (6)	0.0382 (6)	0.0018 (5)	0.0102 (5)	−0.0005 (5)
C9	0.0418 (6)	0.0334 (6)	0.0368 (6)	0.0006 (5)	0.0111 (5)	0.0014 (4)
C10	0.0487 (7)	0.0356 (6)	0.0466 (7)	−0.0034 (5)	0.0134 (5)	−0.0069 (5)
C11	0.0556 (7)	0.0409 (7)	0.0505 (7)	0.0012 (6)	0.0229 (6)	−0.0074 (5)
C12	0.0444 (6)	0.0449 (7)	0.0455 (6)	0.0015 (5)	0.0187 (5)	0.0046 (5)
C13	0.0456 (7)	0.0434 (7)	0.0494 (7)	−0.0079 (5)	0.0131 (5)	−0.0063 (5)
C14	0.0490 (7)	0.0380 (7)	0.0451 (6)	−0.0019 (5)	0.0165 (5)	−0.0068 (5)
C15	0.0492 (8)	0.0726 (11)	0.0883 (11)	−0.0098 (7)	0.0293 (8)	0.0025 (8)

### Geometric parameters (Å, °)

**Table:**

O1—C2	1.3509 (15)	C5—H5	0.9300
O1—H1	0.8200	C6—H6	0.9300
O2—C8	1.2303 (14)	C7—H7	0.9300
O3—C12	1.3695 (14)	C8—C9	1.4839 (16)
O3—C15	1.4248 (17)	C9—C14	1.3870 (16)
N1—C7	1.2773 (16)	C9—C10	1.3997 (16)
N1—N2	1.3780 (14)	C10—C11	1.3759 (17)
N2—C8	1.3604 (15)	C10—H10	0.9300
N2—H2	0.9000	C11—C12	1.3854 (17)
C1—C6	1.3974 (17)	C11—H11	0.9300
C1—C2	1.4085 (17)	C12—C13	1.3864 (17)
C1—C7	1.4538 (16)	C13—C14	1.3805 (17)
C2—C3	1.3891 (17)	C13—H13	0.9300
C3—C4	1.3750 (19)	C14—H14	0.9300
C3—H3	0.9300	C15—H15A	0.9600
C4—C5	1.384 (2)	C15—H15B	0.9600
C4—H4A	0.9300	C15—H15C	0.9600
C5—C6	1.3772 (18)		
			
C2—O1—H1	109.4	O2—C8—N2	121.27 (11)
C12—O3—C15	117.01 (11)	O2—C8—C9	121.51 (10)
C7—N1—N2	118.44 (11)	N2—C8—C9	117.19 (10)
C8—N2—N1	117.41 (10)	C14—C9—C10	118.34 (11)
C8—N2—H2	123.9	C14—C9—C8	117.39 (10)
N1—N2—H2	117.6	C10—C9—C8	124.13 (10)
C6—C1—C2	118.30 (11)	C11—C10—C9	120.40 (11)
C6—C1—C7	119.64 (11)	C11—C10—H10	119.8
C2—C1—C7	122.06 (11)	C9—C10—H10	119.8
O1—C2—C3	117.92 (11)	C10—C11—C12	120.42 (11)
O1—C2—C1	122.26 (11)	C10—C11—H11	119.8
C3—C2—C1	119.82 (12)	C12—C11—H11	119.8
C4—C3—C2	120.29 (13)	O3—C12—C11	116.36 (11)
C4—C3—H3	119.9	O3—C12—C13	123.72 (11)
C2—C3—H3	119.9	C11—C12—C13	119.92 (11)
C3—C4—C5	120.86 (12)	C14—C13—C12	119.39 (11)
C3—C4—H4A	119.6	C14—C13—H13	120.3
C5—C4—H4A	119.6	C12—C13—H13	120.3
C6—C5—C4	119.20 (12)	C13—C14—C9	121.52 (11)
C6—C5—H5	120.4	C13—C14—H14	119.2
C4—C5—H5	120.4	C9—C14—H14	119.2
C5—C6—C1	121.51 (12)	O3—C15—H15A	109.5
C5—C6—H6	119.2	O3—C15—H15B	109.5
C1—C6—H6	119.2	H15A—C15—H15B	109.5
N1—C7—C1	119.60 (11)	O3—C15—H15C	109.5
N1—C7—H7	120.2	H15A—C15—H15C	109.5
C1—C7—H7	120.2	H15B—C15—H15C	109.5

### Hydrogen-bond geometry (Å, °)

**Table:**

*D*—H···*A*	*D*—H	H···*A*	*D*···*A*	*D*—H···*A*
N2—H2···O2^i^	0.90	2.18	3.0112 (15)	153
O1—H1···N1	0.82	1.90	2.6171 (14)	146

Symmetry codes: (i) *x*, *y*+1, *z*.
